# Innate Immunity to Mucosal *Candida* Infections

**DOI:** 10.3390/jof3040060

**Published:** 2017-10-31

**Authors:** Akash Verma, Sarah L. Gaffen, Marc Swidergall

**Affiliations:** 1 Division of Rheumatology and Clinical Immunology, University of Pittsburgh, Pittsburgh, PA 15261, USA; akash.verma@pitt.edu (A.V.); sarah.gaffen@pitt.edu (S.L.G.); 2 Division of Infectious Diseases, Department of Medicine, Los Angeles Biomedical Research Institute at Harbor-UCLA Medical Center, Torrance, CA 90502, USA

**Keywords:** oropharyngeal candidiasis, vulvovaginal candidiasis, epithelial cells, antimicrobial peptides, alarmins, IL-17, innate T cells, antifungal immunity

## Abstract

Mucosal epithelial tissues are exposed to high numbers of microbes, including commensal fungi, and are able to distinguish between those that are avirulent and those that cause disease. Epithelial cells have evolved multiple mechanisms to defend against colonization and invasion by *Candida* species. The interplay between mucosal epithelial tissues and immune cells is key for control and clearance of fungal infections. Our understanding of the mucosal innate host defense system has expanded recently with new studies bringing to light the importance of epithelial cell responses, innate T cells, neutrophils, and other phagocytes during *Candida* infections. Epithelial tissues release cytokines, host defense peptides, and alarmins during *Candida* invasion that act in concert to limit fungal proliferation and recruit immune effector cells. The innate T cell/IL-17 axis and recruitment of neutrophils are of central importance in controlling mucosal fungal infections. Here, we review current knowledge of the innate immunity at sites of mucosal *Candida* infection, with a focus on infections caused by *C. albicans*.

## 1. Introduction

Mucosal candidiasis is a significant problem in both immunocompetent and immunocompromised individuals [1]. Vulvovaginal candidiasis (VVC) is equally common in women who are immunocompetent and immunocompromised, while oropharyngeal candidiasis (OPC) causes significant morbidity in patients who are immunocompromised due to AIDS, neutropenia, diabetes mellitus, or the use of immunosuppressive drugs [2,3]. The development of a mucosal *Candida* infection is usually attributed to the disturbance of the balance between fungal colonization and changes in the host environment. Most episodes of VVC and OPC are caused by *Candida albicans*, a commensal dimorphic fungal organism of the mouth, gastrointestinal, and lower female reproductive tracts [4,5]. Healthy individuals have a protective *Candida*-specific mucosal immunity. These antifungal mechanisms are comprised of numerous components that act in concert to limit fungal invasion, proliferation, and prevent disease. Given the increasing prevalence of mucosal *Candida* infections, it is important to understand the processes that occur during host-*Candida* interactions, in particular, the interplay of soluble host factors and the cellular crosstalk between hematopoietic and non-hematopoietic cells. This review will provide an overview of various host cell types that contribute to the innate mucosal immune response.

## 2. Epithelial Cells: Not Just Physical Barriers

The epithelium separates the host from the environment and provides the first line of defense against pathogens. Traditionally considered as a physical barrier that prevents infection, it is now appreciated that epithelial structures also have direct anti-microbial activity and actively respond to pathogens with a tissue-specific immune defense program [6]. The release of inflammatory mediators from epithelial cells is a critical step for the generation of protective host responses, including recruitment of inflammatory leukocytes and the generation of host defense peptides (HDPs) [7]. In general, invading *C. albicans* cells induce a strong antifungal response in epithelial cells by triggering release of proinflammatory cytokines and chemokines that serve to recruit leukocytes [8,9]. During mucosal *C. albicans* colonization and induction of disease, the fungus adheres to, and invades epithelial cells, causing epithelial cell damage and the release of alarmins [10]. Although mucosal epithelial cells express a variety of pattern recognition receptors (PRRs) that can potentially recognize *C. albicans* [11], the underlying mechanism of fungal recognition is incompletely understood. In addition to canonical PRRs, epithelial cells express receptors, such as the epidermal growth factor receptor (EGFR), human epidermal growth factor receptor 2 (HER2), and E-cadherin that can recognize *C. albicans* hyphae and initiate fungal invasion mechanisms [12,13,14]. Fungal-epithelial interactions induce specific host signaling pathways and epithelial responses [15,16,17], which are also discussed in detail elsewhere [18,19].

### 2.1. Soluble Factors Released during Candida Epithelial Infection: Cytokines, Chemokines and Alarmins 

Epithelial cells respond to *C. albicans* invasion by releasing a specific profile of cytokines [20] that recruit, activate, and differentiate immune cells. This profile includes proinflammatory cytokines, such as interleukin (IL)-1α/β, IL-6, granulocyte-colony stimulating factor (G-CSF), granulocyte-macrophage colony-stimulating factor (GM-CSF), and tumor necrosis factor-α (TNF-α), as well as the chemokines chemokine (C-X-C motif) ligand 8 (CXCL8/IL-8), chemokine (C-C motif) ligand 20 (CCL20) and RANTES [9,15,20,21,22]. Thus, the epithelial cytokine/chemokine profile plays a major part in host defense against invading fungi and provide significant insights into how fungal infections are controlled at mucosal surfaces.

T cell-derived cytokines also play vital roles in epithelial immunity against *Candida* infection, particularly those produced by the IL-17-secreting “Type 17” subset. In mice lacking the IL-17 receptor (IL-17RA^KO^) mucosal expression of murine β-defensin 3 (BD3, encoded by *Defb3*), alarmin S100A8/A9 (Calprotectin) and CCL20 are impaired during oral *C. albicans* infection [23]. Furthermore, IL-17 and IL-22 (also produced by Type 17 cells) cooperatively enhance expression of HDPs by keratinocytes [24,25]. The release of proinflammatory cytokines and chemokines results in the recruitment of neutrophils, key cells for oral mucosal anti-*Candida* immunity [26]. Although, neutrophils are indispensable for the maintenance of mucosal immunity during OPC; these cells can cause pathological inflammation which may be responsible for most of the signs and symptoms of VVC [27,28].

*Candida* species (spp.) induce epithelial cell damage during infection, leading to production of “alarmins” [14,29]. Alarmins alert the immune system of tissue damage following trauma or infection by promoting recruitment and activation of innate immune cells [30,31]. The alarmin family comprises structurally distinct endogenous mediators, including the S100 proteins, heat shock proteins, and nucleosomes [30]. When epithelial cells are damaged, they release alarmins, such as IL-1, S100A8/S100A9 (calprotectin) [27,28,32,33]. S100 alarmins are produced by oral and vaginal epithelial cells, and abundantly by neutrophils [27,34,35]. They are sufficient but not necessary to stimulate polymorphonuclear neutrophil (PMN) migration during VVC [36]. During OPC S100A8 and S100A9 are also strongly induced [23]. Although *C. albicans* triggers more epithelial cell damage than other *Candida* spp., the extent of damage varies among *C. albicans* strains, leading to differences in alarmin production [29,37].

Cellular crosstalk of different cell types is essential in host defense during fungal infection. Beside epithelial cells dermal fibroblast enhance the skin antimicrobial defense during *Candida* infection upon activation through Toll-like receptor 2 (TLR2) and IL-1β secretion [38,39]. Crosstalk between epithelial-derived IL-1α, endothelial cells and neutrophils is required to maintain immunity during oral mucosal *C. albicans* infection. Oral epithelial cells respond to damage induced by *C. albicans* by releasing IL-1α, which stimulates the production of G-CSF on endothelial cells, a key trigger of emergency granulopoiesis [40]. Granulopoiesis and neutrophil mobilization is critical to meet the rapidly increasing demand for neutrophils in the infected tissue if microbial infection cannot be controlled locally [41]. Thus, the interplay of soluble non-hematopoietic host factors and hematopoietic cells is crucial to prevent mucosal disease.

In patients with OPC, *C. albicans* is frequently isolated in conjunction with other *Candida* spp., such as *C. glabrata* or *C. tropicalis* [42,43]. These non-*C. albicans* species (NACs) bind to *C. albicans* hyphae to establish colonization and invasion of the oral epithelium, leading to greater mucosal disease [44]. In isolation, NACs rarely cause OPC and consequently induce a much weaker host inflammatory response. Although *C. glabrata* stimulates epithelial cells to release more GM-CSF compared to *C. albicans*, this spp. does not stimulate proinflammatory cytokines such as IL-1α or CXCL8/IL-8 [45,46]. Furthermore, *C. tropicalis* and *C. parapsilosis* do not induce G-CSF, GM-CSF and IL-6 [47] suggesting that epithelial surfaces respond with specific innate immune pattern to different invading or colonizing *Candida* spp. depending on morphology and host cell damage capacity.

### 2.2. Antifungal Activity and the Propagation of an Inflammatory Response: Host Defense Peptides

**Figure 1:**
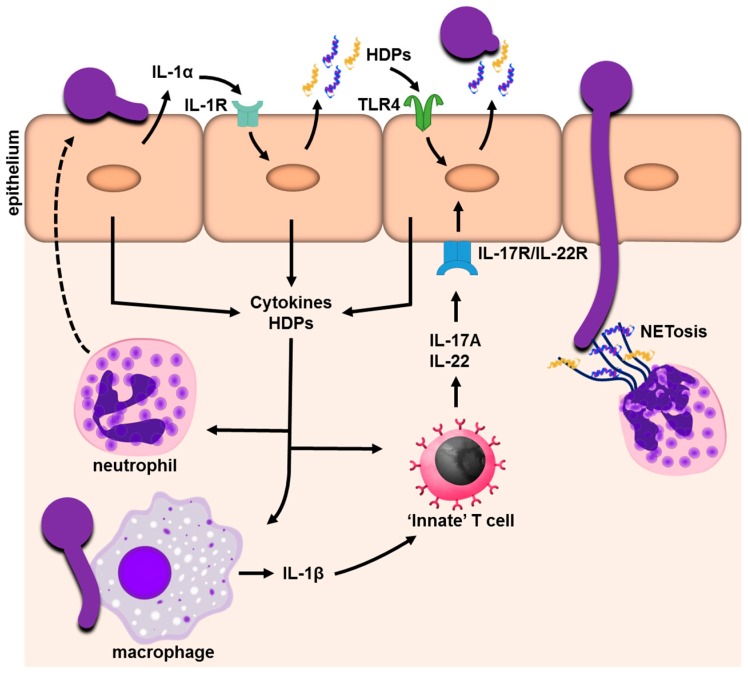
Simplified schematic of the epithelial IL-1-HDP network during *C. albicans* mucosal infection. Upon invasion (receptor-mediated endocytosis and active penetration), *C. albicans* activates epithelial cells to release chemokines, cytokines, and the alarmin IL-1. The cytokine profile will recruit and activate other immune cells, e.g., neutrophils. While neutrophils are capable of phagocytosing *Candida* yeast, the hyphal forms trigger the release of neutrophil extracellular traps (NETs). Epithelial-derived IL-1 binds to the IL-1 receptor (IL-1R) to boost the inflammatory response, including the release of host defense peptides (HDPs). Solid arrows indicate the induction/release of host factors. Dashed line indicates neutrophil recruitment.

The release of HDPs and alarmins is an essential element of the initial epithelial antifungal response [7,37,48,49]. Besides having direct antimicrobial activity, HDPs play an important role in orchestrating the innate immune response by promoting chemotaxis either in a direct and/or indirect manner [50,51]. Although HDPs differ in structure and amino acid composition, they all exhibit broad spectrum activity against microbial pathogens. In the antimicrobial peptide database, 61 human HDPs are listed as being fungicidal [52]. The major HDPs in the oral cavity of humans are the β-defensin family, cathelicidin (LL-37), and histatins [53,54]. β-defensins are expressed by human epithelial cells [55]. In the oral cavity, they have been found in buccal mucosa, gingiva, and tongue epithelium along with salivary glands [56]. The cathelicidin LL-37 is expressed in inflamed gingival tissues, buccal mucosa, and the tongue epithelium [57]. HDPs target the cell wall/membrane of microorganisms to form pores, leading to cytoplasmic membrane dysfunction and ATP/ion release [58]. In addition to interacting with extracellular targets, some HDPs, such as histatin 5 or truncated forms of LL-37, have intracellular targets, including mitochondria [59,60]. The antifungal mechanisms of these HDPs are discussed in detail elsewhere [7,49,59]. Intriguingly, many fungal pathogens including *C. albicans* have evolved mechanisms to evade HDPs. These include the secretion of fungal decoy proteins, proteinases, efflux pumps, and stress response signaling pathways [61,62,63,64,65,66]. Some HDPs are constitutively released, while others are released in response to fungal infection and or the activation of specific host cell receptors (Figure 1) [7,67]. Due to their antifungal activity, HDPs have clear clinical potential. Nonetheless, HDPs can be toxic at high concentrations [68], their expression is, hence, tightly regulated [69]. This is clearly an important area of inquiry, as there are still many aspects of these molecules that are poorly understood. The antifungal mechanisms of these HDPs have been also been reviewed in [7,49,59].

In addition to having broad spectrum antimicrobial activity, some HDPs can act as chemokines to recruit immune cells and modulate cytokine release [70]. The cathelicidins, LL-37 and CRAMP (Cathelicidin-related Antimicrobial Peptide; the mouse homolog of human LL-37), recruit and activate innate immune cells such as dendritic cells (DCs) and, as a result, promote adaptive immune responses. Human neutrophil peptides (hNPs) induce IL-8 secretion by epithelial cells to attract leukocytes [71], while the human β defensin 3 (hBD3) attracts monocytes by binding to chemokine receptor 2 (CCR2) [72]. hBD2 and its ortholog mouse BD3 can bind to the receptor CCR6, found on neutrophils and Type 17 cells, and may serve as a chemotactic agent for lymphocytes and neutrophils [73,74,75]. At physiological concentrations, some HDPs can also stimulate the production of chemokines, such as CXCL1, IL-8/CXCL8, CCL2, CCL4, RANTES, and CCL20 [76,77].

During OPC, there is a large increase in the levels of HDPs such as BD*3* [23]. Mice deficient in IL-17 receptor A (IL-17RA) or IL-17RC fail to induce HDPs in the epithelium in response to *C. albicans*, a major cause of susceptibility to OPC in mice and humans [23,78,79]. Mice with an oral epithelial cell specific deletion of IL-17RA have reduced expression of BD3 in the oral mucosa and increased susceptibility to OPC. This increased susceptibility is phenocopied by deletion of BD3 [79]. Mice deficient in murine β-defensin 1 (mBD1) are also more susceptible to OPC [80]. In addition to its direct antifungal activity, mBD1 recruits neutrophils to sites of mucosal fungal infection and regulates expression of other HDPs including mBD2 and LL-37 (CRAMP in mice) [80]. Patients with Th17 defects due to a dominant-negative mutation in signal transducer and activator of transcription 3 (STAT3), a disease known as Job’s syndrome, are highly susceptible to mucosal *C. albicans* infections. Strikingly, these patients show a marked impairment in salivary HDPs β-defensin 2 and histatins [81]. Collectively these findings demonstrate the importance of epithelial cell-derived HDPs in the host defense against OPC.

The role of HDPs in VVC is incompletely understood, but they are likely to be important players here as well. In vitro, human vaginal epithelial cells secrete hBD1 and hBD2 when stimulated with *C. albicans* [82]. In a mouse model of VVC, β-defensin 2 increased after *C. albicans* infection [83]. However, estrogen exhibits immunomodulatory effects by decreasing HDP expression and modulating PRR expression [84]. Thus, additional studies to elucidate the role of HDPs in VVC are needed.

In the gut, commensal bacteria regulate HDP expression [85]. Anaerobic bacteria inhibit *C. albicans* colonization of the gut in mice by increasing the expression of LL-37 through the transcription factor HIF-1α [86,87]. In inflammatory bowel disease (IBD), a normally commensal, or even mutualistic microbial community, turns delinquent, and subsequently promotes an ongoing inflammatory response [88]. The resulting intestinal inflammation is believed to be attributed to an aberrant immune response against commensal gut microbes, but the exact pathogenesis remains unclear [89]. Therefore, an altered composition of the gut microbiota, e.g., antibiotic treatment, can influence and destabilize epithelial defenses by decreasing HDP expression leading to fungal colonization and/or proliferation. In addition to bacterial dysbiosis, prolonged oral treatment with antifungals leads to alterations of commensal fungal populations that can influence local and peripheral immune responses and enhance relevant disease states [90].

Other reported activities of defensins include the activation of the classical complement pathway via both C1q-dependent and independent mechanisms [91,92]. In contrast, the defensin human neutrophilic peptide 1 (HNP1) inhibits both the classical and lectin pathways of complement [93]. Furthermore, HDPs play a critical role in promoting initiation, polarization and amplification of adaptive immunity by (i) chemotaxis of immature dendritic cells (iDC), (ii) modulation of lymphocyte activity, (iii) direct iDC activation via TLR4, and (iv) the generation of primed iDCs with enhanced antigen uptake and presentation capacity [70,94]. Collectively, HDPs are major antifungal defense molecules with direct antimicrobial activity, as well as immunomodulatory functions, which contribute to the antifungal machinery on mucosal surfaces.

## 3. Hematopoietic Cell-Mediated Innate Immunity in *Candida* Infections

In addition to epithelial cells, mucosal sites are enriched with a network of hematopoietic cells that bolster antifungal barrier defenses. These leukocytes are activated rapidly by cues from epithelial cells and employ a diverse array of mechanisms to limit pathogen invasion. In this section, we summarize the important contributions of innate immune cells in mucosal *Candida* infections.

### 3.1. γδ T Cells, Innate TCRαβ^+^ Cells, and Type-3 Innate Lymphoid Cells: Cellular Sources of IL-17 

The cytokine IL-17 is an integral component of host antifungal barrier immunity [95]. IL-17 elicits anti-*Candida* responses through disparate mechanisms. The cytokine (i) mobilizes neutrophils to the site of infection *via* the release of CXC chemokines, (ii) prompts non-hematopoietic cells to secrete HDPs including β-defensins, S100A proteins and histatins, and (iii) reinforces the proinflammatory cascade by synergizing with cytokines such as IL-1 and TNFα. Indeed, humans with genetic defects in the IL-17 signaling pathway or immune pathways that shape Th17 responses exhibit severe susceptibility to mucosal *Candida* infections [96,97,98]. Moreover, neutralizing antibodies against IL-17 predispose individuals to chronic mucocutaneous candidiasis (CMC), commonly seen in *AIRE*-deficient patients [99,100] and occasionally in individuals undergoing anti-IL-17 biologic therapy for autoimmunity [101]. IL-17 is made by conventional T_H_17 cells, but additionally multiple innate lymphocyte subsets produce the cytokine during the early stages of infection.

#### 3.1.1. γδ T Cells 

These cells are major early sources of IL-17 during mucosal infections [102]. γδ T cells are thymically-derived and express an unconventional γδ T cell receptor (TCR). The precise molecular events that program IL-17^+^ γδ T cells are not fully understood, but the development of this γδ T cell-subset occurs in waves during the fetal and neonatal stages and these cells seed the peripheral sites with fairly restricted TCR Vγ specificity [102,103]. Once in the periphery, these cells possess the capacity to respond rapidly to invading pathogens and secrete large quantities of IL-17 (or other cytokines) without a requirement for antigen presentation.

Several seminal studies have demonstrated the vital contributions of IL-17^+^ γδ T cells in limiting *C. albicans* invasion at barrier interfaces. In the skin, γδ T cells are activated by signals from the nervous system [104]. *C. albicans* activates directly cutaneous sensory neurons to release a neuropeptide, calcitonin gene-related peptide (CGRP) that influences tissue resident dendritic cells to secrete IL-23 [104]. This potentiates swift activation and proliferation of IL-17-producing γδ T cells and eventual clearance of infection in the skin, thus linking the neuronal system to immunity against fungal infections. How fungal PAMPs activate sensory neurons in the skin is unclear, however, during fungal osteoinflammation *C. albicans* activates neurons via the dectin-1-TRP channel axis, leading to CGRP production [105]. Therefore it is likely that the fungal cell wall component β-glucan stimulates cutaneous sensory neurons during invasion to enhance innate immune responses in deeper tissues.

In the oral mucosa, γδ T cells constitute an innate source of IL-17 following *C. albicans* infection [106]. In the eye, IL-17^+^ γδ T cells generated in response to ocular commensal bacteria provide broad non-specific immunity (heterologous immunity) against IL-17-sensitive pathogens such as *C. albicans* [107]. Hence, their ‘innate-mode’ of activation makes γδ T cells vital for fortifying barrier defenses.

#### 3.1.2. TCRαβ^+^ Cells 

Barrier sites, such as the oral mucosa and skin, are lined with a population of TCRαβ^+^ cells that exhibit innate properties [104,106]. Much like the IL-17^+^ γδ T cells, these innate-acting TCRαβ^+^ cells express IL-17 and expand rapidly at the sites of *C. albicans* infection without engagement of their TCR [106,108]. A property that distinguishes this subset of T cells from conventional antigen-specific T_H_17 cells is their lack of dependence on canonical fungal pattern recognition receptors including Dectin-1, CARD9, and TLR2 [108,109]. In the oral cavity, innate TCRαβ^+^ cell proliferation is instead reliant on the fungal pore-forming toxin Candidalysin [22,108]. Epithelial cell damage by Candidalysin prompts the release of IL-1α and IL-1β, which, in turn, drives TCRαβ^+^ cell-expansion through T cell-intrinsic and extrinsic mechanisms. In the skin, CD8^+^ T cells specific for skin commensal bacteria provide heterologous immunity against *C. albicans* [110]. These cells are functionally analogous to the IL-17^+^ γδ T cells in the eye and hints at the existence of conserved defense mechanisms at the mucosae. Hence, TCRαβ^+^ cells can be co-opted to operate in an innate manner at barrier interfaces. The increased flexibility in effector functions perhaps represents an effective evolutionary approach devised by the host to counter invasive pathogens.

#### 3.1.3. Innate Lymphoid Cells (ILCs) 

ILCs are lymphocytes that do not express rearranged antigen-specific receptors and, hence, are present in Rag-deficient mice that lack conventional T and B cells [111]. ILCs are classified into three major groups based on their capacity to produce T_H_1-, T_H_2-, or T_H_17-associated cytokines. RORγt^+^ ILCs (ILC3s) have been reported to contribute to early protection against *C. albicans* infection by expressing IL-17 in the oral mucosa [112]; however, Rag1^−/−^ mice are still susceptible to oral candidiasis, so their contribution is insufficient to protect against acute infection [106,113,114]. ILC3s have also been reported at other barrier surfaces such as the gut, lungs, eyes, and skin [111], however, their role in limiting fungal pathogens at these sites is unclear. Another area of inquiry is the influence of ILCs on adaptive responses. Functional contribution of ILC3s has been reported at day 7 of acute oral *Candida* infection wherein these cells may strengthen mucosal defenses against future pathogen encounters [112].

An additional role of ILC3s may be in the maintenance of epithelial barrier integrity. IL-22 is another signature cytokine released by ILC3s that promotes epithelial regeneration, especially in the gut, and also the production of HDPS such as β-defensins, RegIIIγ, S100A proteins, and lipocalin during infection [115]. Indeed, IL-22 is crucial for limiting *C. albicans* growth in the gut [116]. In experimental gastric candidiasis, IL-22^−/−^ mice display higher fungal burden and impaired barrier integrity, as opposed to wild-type controls. Furthermore, a protective role for IL-22 has been described in VVC [117].

In summary, the host barrier surfaces appear to have crafted multiple redundant strategies to rapidly secrete type-17 cytokines and thus limit pathogenic *C. albicans* invasion.

### 3.2. Interaction of Candida with Neutrophils

Neutrophils are indispensable for host defense against fungal infections and are typically the first responding leukocytes to be mobilized in large numbers to the infected site. While the contributions of neutrophils have been best studied in the context of *C. albicans* [26,40], reports have described their importance in controlling non-*C. albicans* species such as *C. glabrata* [118], *C. tropicalis* [119], and *C. parapsilosis* [120]. Neutrophils aggressively ingest and destroy fungal particles through phagocytosis. Two disparate phagocytic killing mechanisms have been reported [121]. First, unopsonized *C. albicans* are recognized by complement receptor 3 (CR3) and fungal killing is dependent on the CARD9 pathway. Second, opsonized yeasts are internalized *via* Fcγ receptors and pathogen killing is achieved through intracellular NADPH activity. In addition to phagocytosis, neutrophils possess a plethora of other fungicidal weapons in their arsenal [122]. The granulocytes release neutrophil extracellular traps (NETs), reactive oxygen species (ROS), and secrete soluble mediators such as HDPs, proteases and proinflammatory cytokines to further facilitate pathogen clearance. The importance of neutrophil-fungicidal effector functions has been confirmed in human studies. Individuals with a mutation in the adaptor protein CARD9 exhibit high susceptibility to mucosal *Candida* infections [123,124,125]. One of the leading causes for this predisposition appears to be a defective microbicidal activity of CARD9-deficient neutrophils [126].

Intriguingly, the fungicidal strategy used by neutrophils may be dictated by pathogen size [127]. Smaller fungal particles such as *Candida* yeasts are rapidly internalized and destroyed in phagolysosomes. In contrast, larger non-ingestible hyphal segments are ensnared by a web of nucleic acids, histones, and antimicrobial proteins that neutrophils extrude upon pathogen contact, a phenomenon called NETosis or NET-attack [127]. Another consequence of ‘NET-attack’ is unmasking of the fungal PAMPs on *C. albicans* cell wall that direct an appropriate innate immune response to the pathogen. In disseminated candidiasis, NET-attack causes exposure of β-glucan moieties on fungal surfaces, thus augmenting early recognition by Dectin-1 [128]. Larger fungal particles can also be sensitive to extracellular ROS from neutrophils that further aids in resolution of infection [129]. In addition to directly engaging fungi, neutrophils employ indirect mechanisms to starve pathogens of vital nutrients. Specifically, the release of Calprotectin (S100A8/9) sequesters trace metals like zinc and manganese, restraining *C. albicans* growth [35,130]. Thus, their diverse ‘bag of tricks’ make neutrophils a vital component of innate antifungal defenses.

### 3.3. Candida and Mononuclear Phagocytes

Dendritic cells, macrophages, and monocytes collectively make up the mononuclear phagocyte arm of the innate immune system. Mononuclear phagocytes readily internalize and kill ingested microbes by ROS-dependent and independent processes [131]. These cells express innate pattern recognition receptors such as C-Type Lectin Receptors (CLRs) and TLRs that sense fungal pathogens. In response to tissue invasion, these cells trigger a proinflammatory cascade of cytokines and chemokines. In acute dermal candidiasis, a subset of skin-resident dendritic cells secrete IL-23, which in turn activates IL-17-driven antifungal responses [104]. In addition to strengthening early defenses, mononuclear phagocytes form an important bridge to the adaptive phase of immunity. At mucosal sites, dendritic cells take up and process invading *C. albicans.* The phagocytes then traffic to secondary lymph nodes where they polarize anti-fungal T cells to the T_H_17 lineage [113,132].

Another intriguing facet of monocyte biology is the ability to imprint immunological memory of previously encountered microbial antigens, a concept known as ‘trained immunity’ [133,134]. Repeated exposure with the fungal PAMP β-glucan induces epigenetic and metabolic modifications in monocytes, and may thereby prime innate cells to respond more effectively to subsequent *C. albicans* (and other) infections [135,136]. This finding illustrates that the line between innate and adaptive immune responses is more blurry than typically viewed.

## 4. Conclusions

The mucosal immune response is a meticulously regulated system of opposing pro- and anti-inflammatory mediators of various cellular sources to balance immune homeostasis. The precise antifungal innate network distinguishes between commensal and pathogenic forms of *Candida* and turns on the innate immune machinery to prevent fungal infections in healthy individuals. An imbalance of this crosstalk between hematopoietic and non-hematopoietic cells results in fungal commensal proliferation leading to disease. The immune defense of barrier sites has remained a seriously understudied topic. Future studies designed to further our understanding of mucosal immunity homeostasis and activation during fungal colonization and proliferation may lead to novel therapeutic approaches to fighting infection.
